# The Nutrinet-Santé Study: a web-based prospective study on the relationship between nutrition and health and determinants of dietary patterns and nutritional status

**DOI:** 10.1186/1471-2458-10-242

**Published:** 2010-05-11

**Authors:** Serge Hercberg, Katia Castetbon, Sébastien Czernichow, Aurélie Malon, Caroline Mejean, Emmanuelle Kesse, Mathilde Touvier, Pilar Galan

**Affiliations:** 1 Unité de Recherche en Epidémiologie Nutritionnelle; UMR U 557 Inserm/U 1125 Inra/CNAM/Université Paris 13; CRNH Idf, 74 rue Marcel Cachin, F-93017, Bobigny, France; 2 Unité de Surveillance et d'Epidémiologie Nutritionnelle; Institut de Veille Sanitaire, Université Paris 13; F-93017, Bobigny, France; 3 Département de Santé Publique, Hôpital Avicenne, F-93017 Bobigny, France

## Abstract

**Background:**

Nutrition-related chronic diseases such as cardiovascular diseases and cancer are of multiple origin, and may be due to genetic, biologic, behavioural and environmental factors. In order to detangle the specific role of nutritional factors, very large population sample cohort studies comprising precisely measured dietary intake and all necessary information for accurately assessing potential confounding factors are needed. Widespread use of internet is an opportunity to gradually collect huge amounts of data from a large sample of volunteers that can be automatically verified and processed. The objectives of the NutriNet-Santé study are: 1) to investigate the relationship between nutrition (nutrients, foods, dietary patterns, physical activity), mortality and health outcomes; and 2) to examine the determinants of dietary patterns and nutritional status (sociological, economic, cultural, biological, cognitive, perceptions, preferences, etc.), using a web-based approach.

**Methods/design:**

Our web-based prospective cohort study is being conducted for a scheduled follow-up of 10 years. Using a dedicated web site, recruitment will be carried out for 5 years so as to register 500 000 volunteers aged ≥ 18 years among whom 60% are expected to be included (having complete baseline data) and followed-up for at least 5 years for 240 000 participants. Questionnaires administered via internet at baseline and each year thereafter will assess socio-demographic and lifestyle characteristics, anthropometry, health status, physical activity and diet. Surveillance of health events will be implemented via questionnaires on hospitalisation and use of medication, and linkage with a national database on vital statistics. Biochemical samples and clinical examination will be collected in a subsample of volunteers.

**Discussion:**

Self-administered data collection using internet as a complement to collection of biological data will enable identifying nutrition-related risks and protective factors, thereby more clearly elucidating determinants of nutritional status and their interactions. These are necessary steps for further refining nutritional recommendations aimed at improving the health status of populations.

## Background

Numerous clinical, physiopathological and epidemiological studies have underlined the important role of nutritional factors in decreasing or increasing the incidence of chronic diseases such as cardiovascular diseases, some cancers, diabetes, obesity, hypertension and osteoporosis [1,3]. These pathologies represent a heavy burden for public health in France and other industrialized countries due to the high mortality rate and high costs associated with them [3,4]. Identifying nutrition-related risk and protective factors is a necessary step for further improving nutritional recommendations aimed at benefiting the health status of populations. These diseases have multiple origins, involving genetic, biologic, behavioural and environmental factors. In order to assess the specific role of nutritional factors involved, very large population sample cohort studies, comprising precisely measured dietary intake and all necessary information for effectively controlling potential confounding factors, are needed [2,5].

Indeed, the use of internet offers a unique opportunity for gaining access to a vast sample of volunteers. France ranks in first place among European countries in use of the internet [6]. In November 2009, 34.7 million French citizens (about 65% of the population over 11) had been connected to the internet during the previous month irrespective of the connection site (home, work, public or private location) [7,8]. One French internet user out of 4 is over 55 years of age and 29% of users belong to low socio-professional categories [7]. Thus, the profile of internet users would reflect an accurate representation of the different age groups (especially seniors), socio-professional categories and regions. Indeed, the use of internet offers the possibility of automatically gaining access to immense samples of volunteers as well as gradually collecting, saving and processing huge amounts of information at reduced logistic burdens and costs.

The present paper describes the methodology of the NutriNet-Santé Study, a large web-based prospective study launched in France in May 2009.

## Methods/Design

### Aim

The objectives of the NutriNet-Santé Study are: 1) to investigate the relationship between nutrition (nutrients, foods, dietary patterns, physical activity, nutritional status) and health outcome (incidence of cardiovascular diseases, cancers, diabetes, obesity, hypertension, mortality, etc.); and 2) to study the role of various determinants (sociological, economic, cultural, biochemical, cognitive, perception, food preferences, etc.) of dietary patterns and nutritional status, and their interactions.

### Study design

Our web-based prospective cohort study is scheduled for a 10-year follow-up. Using a dedicated web site, recruitment will be carried out for 5 years. The study is being implemented in a general population targeting internet-using adult volunteers aged 18 years or more. After registration, participants will be included and then followed via a web site specifically created for this purpose. Questionnaires and forms are ready-made and can be directly filled in online using a secure HTLM interface where all security conditions of data and personal information are provided (interface secured by an SSL certificate 128 bit).

In order to be considered as included, participants (or "Nutri-netters") must completely fill in an initial set of questionnaires aimed at assessing the following: diet (3 random records of 24 h food intake during a two-week period), physical activity, anthropometry, lifestyle and socio-economic conditions and health status. As part of their follow-up schedule, the Nutri-netters will complete this set of questionnaires every year. Moreover, they will receive a monthly automated e-mail informing them of the necessity of completing their files by filling out a new questionnaire (which takes less than 20 min each month) in their personal space on the website of the study. Data will be regularly collected on determinants of food behaviour and nutritional status and on the health status of participants (morbidity, quality of life, etc.). It will also be possible to add to the basic protocol different questionnaires "à la carte" so as to develop ancillary protocols concerning the whole population or subsamples chosen according to a particular phenotype (age, sex, area of residence, health status, etc.).

Moreover, participants will be invited, via the website, to go to one of the specific health centres involved in the study, located in various French cities. During the visit, they will undergo blood and urine sampling (used in part to develop a biobank) and a clinical examination.

Concerning outcome, data concerning the participant's eventual decease and cause of death will be obtained from a national database on mortality of the French population [9]. Health events will be followed up using a declaration by participants on the website and, after collection of medical records, it will be validated by independent committees of experts.

This study was approved by the International Research Board of the French Institute for Health and Medical Research (IRB Inserm n° 0000388FWA00005831) and the "Comité National Informatique et Liberté" (CNIL n° 908450 and n° 909216).

### Recruitment and inclusion

Eligible participants are adults 18 years or older, with access to internet, recruited via different measures begun in May 2009 and which will be ongoing for the 5 years of inclusion. A vast multimedia campaign (television, radio, national and regional newspapers, posters, internet) will call for volunteers by providing details on the study's specific web-site http://www.etude-nutrinet-sante.fr where volunteers can subscribe. A relay of information is being maintained on a large number of websites (national institutions, city councils, private firms, web organisations) A billboard advertising campaign is being regularly updated through professional channels (doctors, pharmacists, dentists, business partners, municipalities, etc.) An additional invitation to participate will be given to participants in the CONSTANCES cohort, another cohort study which will be launched in 2011 (200,000 subjects aged 18 to 70 followed up at the national level at health examination centres of the French Social Security Health Insurance System).

### Sample size

The sample size was calculated to obtain strong power for examining the various chronic disease risk factors of interest. For instance, taking into consideration the selection bias (volunteers) and on the basis of half of the participants being over 45 years of age, 18,000 to 20,000 new cases of cancers, 25,000 to 30,000 new cases of cardiovascular diseases and around 20,000 deaths during the 10 years of follow-up would be expected. The sample size and recruitment of participants of all age groups, at a national level will also enable us to examine the role of various determinants of dietary patterns and nutritional status.

During the five years of recruitment, we plan to enroll 500,000 adults 18-years-old or more, and among them, at least 250,000 participants over 45.

Participation is considered firm once the participants have completely filled in the "initial set" file (basic kit) containing five types of questionnaires (socio-demographic and lifestyle data, health status, diet, physical activity, anthropometry) which must be done in the 21 days following registration. From among the 500,000 registered volunteers, we are targeting a rate of inclusion of 60%, thereby attaining 300,000 persons having complete baseline data. By using the national administrative mortality database, no loss of follow-up can occur in terms of this outcome in the sample of persons included. For the other main outcomes, we foresee a loss rate of 20% among included participants due to loss-to-follow-up and dropouts, leading to a final total of 240,000 participant with complete follow-up.

### Data collection techniques

Once the registration form is completed and the consent form signed (mandatory steps), the participant will receive a confirmatory e-mail providing an individual identification number and password. At this stage, he/she will have 21 days to connect via the "member access" rubric using his/her individual identification number and password. When the participant connects for the first time, dates for the three 24 h dietary records are automatically randomly selected (during a 2-week period) and the participant is notified.

### 1. Information collected at inclusion and every year as part of the "initial set "

The "initial set" file inclusion contains five types of questionnaires: alimentary questionnaires (3 × 24 h recall); socio-demographic and lifestyle questionnaires; health questionnaires; anthropometric questionnaires; and physical activity questionnaires

These have to be filled in within the 21 days following drawing of the dietary survey dates. The order when filling out these questionnaires is at the discretion of the participant, except for the randomly selected 3 days of the dietary survey. General instructions on how to fill out the questionnaires automatically appear when they are opened, are in the form of texts and illustrations. Some questions are programmed to lead the postulant through specific fields, i.e. the answer to a given question may trigger a "jump" which would engender a new and specific question, or it might skip over the next questions depending on the person's answer. For most questions, an answer will trigger the following question. Nevertheless, some questions contain the option "I don't know" or "I don't want to answer". If the participant forgets to click on an answer, an error message appears before the he/she can go on to the next page; it points out the item to be completed. Some questions are subject to control (e.g. a hospitalisation date cannot be posterior to the date of filling in the questionnaire). In such cases, an error message appears pointing out which answer should be corrected.

#### 1. 1. Dietary assessment

At inclusion, the participants complete three 24 h dietary records. The interactive record is designed for self-administration on the internet. It is based on a secured user-friendly interface and includes detailed instructions in several forms (PDF user's guide, video, tips included within the questionnaire, etc.). It relies on a meal-based approach, recording all foods and beverages consumed (nature and quantity) at each time food is eaten: breakfast, lunch, dinner and all other eating occasions. Time and place of each eating occasion is systematically recorded. First, the participant fills in the names of all food items eaten, with 3 possibilities: 1) a food browser in which foods are grouped by category (vegetables, dairy products, etc.) into a classification tree in which the participant browses each branch until reaching the consumed food item; 2) a search engine that accepts spelling errors; and 3) manual typing (in case the food has not been found by the first two methods). For specific foods with potentially high nutrient variability, participants are asked to provide the brand name.

In order to avoid omissions, supervision is integrated at two levels: 1) for each food entered, the software proposes a list of other items usually associated with it (e.g. sugar in coffee); and 2) at the end of the food entry step of each eating occasion, it proposes a reminder of usually consumed items, such as water, bread, sugar, salt, etc.

Next, the participant estimates portion sizes for each food and beverage previously listed using photographs directly included in the computerized interface. These photographs, from a validated picture booklet [10], represent more than 250 foods (corresponding to 1,000 generic foods) proposed in three different portion sizes. Along with the two intermediate and two extreme quantities, there are seven choices of amounts. Instead of using the photographs, the participant can directly enter the quantity consumed in grams or the volume, if known.

Upon completion, the participant declares whether the reported items were fairly representative of his/her usual diet or strongly differed due to specific events (illness, a social event, etc.).

#### 1. 2. Social, demographic, economic and lifestyle questionnaires

Information is being collected concerning marital status, number of children and grandchildren, number and characteristics of family members, current (or last occupied) job, diploma, spouse's professional status and education level, income, tobacco smoking (type, quantity, duration, passive smoking, etc.), alcohol consumption (type, quantity, frequency, etc.)

#### 1.3. Health questionnaires

Information is collected concerning past medical history, past and current use of medication, dietary supplements, familial medical history, causes of death of first-degree relatives (when appropriate) and, for women, obstetrical history, pregnancies, menopausal status, contraception and hormone replacement therapy at menopause when applicable.

#### 1.4. Anthropometric questionnaires

The NutriNet-Santé anthropometric questionnaires include questions dealing with self measurement of current height, weight, hip and waist circumferences, weight history, practice of restrictive diets (type and reason, history), body self-perception.

#### 1.5. Physical and sedentary activity questionnaires

The self-administered questionnaire on physical activity is a web version of the "International Physical Activity questionnaire" (IPAQ) [11]. Physical activity is described according to 3 levels of exercise intensity (walking, moderate or vigorous), frequency of exercising per week (days/week) and daily duration of each performed activity. Following the recommendations of the IPAQ research committee, and using information collected in these 3 categories of energy expenditure, it is possible to estimate weekly energy expenditure in metabolic equivalent expenditure expressed in MET-minutes/week. Moreover, a sedentary life pattern is roughly evaluated by time spent in front of a screen (TV, computer, etc.).

Every year, the participant will once again fill in the basic questionnaire kit and three 24 h dietary records. Questionnaires will be adapted for measuring changes when applicable.

### 2. Additional questionnaires

Between two annual reports, the participant will be asked each month to fill in complementary questionnaires. A semi-quantitative food frequency questionnaire will be implemented 6 month after inclusion. Other questionnaires will enable collecting data on quality of life (SF-36), living conditions, food storage and dietary habits, behavior and restrictions, knowledge of nutritional recommendations, food preferences, etc.

### 3. Information on health status

Data on patient's eventual decease and cause of death will be obtained following the procedure described in the decree 98-37 that authorizes access to the French database on vital statistics [9].

Moreover, via the website, participants are invited to systematically provide information on all major health events. In case of declaration of a health event, medical records will be requested (hospitalization, diagnosis, etc.) to validate the information. If necessary, the medical team of the Nutrinet-Santé Study will collect additional information from the participant's treating physician or the medical structures which he or she frequented. They will collect hospital notes, treatment reports, laboratory reports, etc. Such data will be reviewed by independent committees of experts for validation.

### 4. Biological and clinical assessment and biobank

From a subsample of volunteers, biological samples will be collected for a wide range of laboratory investigations. The methods of sampling and collection of biological samples adopted by the NutriNet-Santé biobank largely reproduce those used by the UK biobank (national ongoing British biobank recruiting 500,000 participants from whom biological samples are collected at 35 different centres [12]).

Participants will be asked to visit the local sample collection centres (LSCC) of the NutriNet-Santé Study specifically set up for blood and urine sampling in each region (about 30 hospital-located centres are participating in the collection; at any given time, 6 centres will be in operation). The contact and the appointments will be managed by a centrally computerized system. The system will function through the internet connexion and will contact study participants on a specially designed website. Individuals will be encouraged to undergo a series of check-ups, including blood cholesterol (total, HDL and LDL), triglycerides and blood glucose levels (a copy of the results will inform them of abnormal values if present). Samples will be collected from 40 participants in each of the 6 LSCC per day, giving a total of 240 participants per day, 40,000 a year and 200,000 participants over the 5-year period.

Blood samples will be collected using vacutainer systems. A total of 43 ml of blood will be collected from each volunteer to be distributed into 5 vacutainers. A urine sample will be collected at the same time. A variety of tubes containing different kinds of anti-coagulants and separators will be used. Anticoagulants such as EDTA K2 and lithium-heparin and the obtained sera will augment the possibility of future use of samples. From each volunteer, blood will be collected into two 9 ml tubes containing EDTA K2, one 9 ml tube containing lithium-heparin and two 8 ml plastic collection tubes. These vacutainers will be used to obtain plasma, serum, buffy coat (for DNA extraction) and red blood cells. The tubes will then be fractionated into sufficient aliquots to serve for future analyses not yet scheduled in the study. In addition, the lithium-heparin tube and plastic tubes containing an inert gel for facilitating separation of blood cell components will be used to prevent potential changes in plasma and serum during the time which elapses from tube centrifugation at local centres to aliquot production at the central processing laboratory.

The set of tubes (blood and urine) collected from each participant will be fractionated into a total of 28 aliquots to be stored at very low temperature (-80°C) at the central laboratory of the biobank.

During the medical visit, in addition to blood collection, a clinical examination will be performed and will include blood pressure, grip test, weight, height, waist and hip perimeters and bio-impedance measurements).

### 5. Ancillary protocols

In the context of ancillary protocols dealing with different themes of scientific interest, it will be possible to include specific questionnaires for the overall population or subsamples chosen according to particular phenotypes (age, sex, region, health status, etc.). Participants will be invited to fill in these questionnaires once they are connected to the study's web site. An interface on the web site will permit the administrators to create research groups and determine whether participants have a profile or a manner of answering questions which falls within certain categories or corresponds to certain selected criteria for grouping participants (e.g. all non-smoking males over 45).

## Discussion

Nutritional epidemiology is of crucial importance when investigating nutrition-health relationships. It provides direct information exposure to certain dietary factors as related to the development of pathologies in everyday life. Use of the internet in the NutriNet-Santé Study is enabling the setting up of a prospective study conducted on a large sample of subjects, with maximum control of confounding factors via detailed phenotyping of subjects [13,14].

The NutriNet-Santé Study, the first international web-based cohort study, will provide an immense depository for phenotypic data, with long-term follow-up. This storage will include different data collected via internet questionnaires (dietary exposure, physical activity, determinants of nutritional status, health events, etc.) and those obtained through data linkage with mortality registries. Moreover, combining this data with biological samples (blood and urine) on which many kinds of analyses can be done (genetic, proteomic, metabolomic, biochemical, etc.) will offer an exceptional resource for validating numerous hypotheses, especially those concerning physiopathological mechanisms, and for evaluating biomarkers and integrating genetic procedures into the analysis of the relationship between nutrition and health.

## Competing interests

The authors declare that they have no competing interests.

## Authors' contributions

SH and PG designed the study; SH, PG, KC, SC, EK, MT and CM participated in the elaboration of the protocol. SH, PG, KC, SC, MT, CM, EK and AM will participate in the acquisition of data and drafted the manuscript.

All authors have read and have approved the final manuscript.

## Pre-publication history

The pre-publication history for this paper can be accessed here:

http://www.biomedcentral.com/1471-2458/10/242/prepub
